# Real-World Prescription Patterns For Patients With Young-Onset Parkinson’s Disease in China: A Trend Analysis From 2014 to 2019

**DOI:** 10.3389/fphar.2022.858139

**Published:** 2022-05-12

**Authors:** Xiao-qin Liu, Xiao-yu Wang, Hui-ming Shen, Wen-yuan Pang, Ming-kang Zhong, Chun-lai Ma

**Affiliations:** ^1^ Department of Pharmacy, Huashan Hospital, Fudan University, Shanghai, China; ^2^ Department of Pharmacy, The Affiliated Suzhou Hospital of Nanjing Medical University, Suzhou Municipal Hospital, Nanjing, China; ^3^ Beijing Prescription Consulting Ltd., Beijing, China; ^4^ Department of Pharmacy, Beijing Tiantan Hospital, Capital Medical University, Beijing, China

**Keywords:** prescribing pattern, young-onset Parkinson`s disease, levodopa, dopamine agonist, levodopa equivalence daily dose

## Abstract

**Introduction** Pharmacotherapy is one of the main treatments for patients with young-onset Parkinson’s disease (YOPD). Although numerous studies on the treatment of YOPD have been published, the real-world prescription patterns of these populations remain unclear in China.

**Methods** A national comprehensive evaluation was performed to reveal the pharmacological treatment patterns in Chinese patients with Parkinson’s disease from 1 January 2014 to 31 December 2019, with patients aged 21–50 years classified as having YOPD for the subgroup analysis. Information on patients and drugs was extracted to analyse the demographic characteristics, prescription patterns, and levodopa equivalent daily dose (LED) during disease progression.

**Results** A total of 1,134 patients with YOPD were included, and the majority were aged 41–50 years. Prescription of L-DOPA/benserazide and pramipexole accounted for more than 30 and 20%, respectively, in each year from 2014 to 2019. There was no difference in prescription patterns in terms of age, sex and geographical areas. Half of the patients with YOPD were on monotherapy, but the proportion decreased from 2016. Correspondingly, the proportion of patients receiving polytherapy increased, especially those who were prescribed more than two anti-Parkinson’s disease drugs. During the disease course, LED showed high variability, which increased over time.

**Conclusion** L-DOPA/benserazide and pramipexole were the most frequently prescribed anti-PD drugs for patients with YOPD in China. There was a slight trend in the transition from monotherapy to polytherapy. LED increased with disease duration. Thus, we provided an overview of the prescription patterns for patients with YOPD in China.

## Introduction

Parkinson’s disease (PD) is a common neurodegenerative disease characterised by a group of neurological symptoms, such as rigidity, slowness, and tremors (Armstrong and Okun, 2020). The prevalence of PD increases with age and is approximately 3.9% in those aged ≥ 50 years in China (Zou et al., 2015; Li et al., 2019). It has been estimated that by 2030, the number of patients with PD in China would increase to 4.94 million and account for more than half of patients with PD worldwide (Calabrese, 2007).

Usually, the median age of onset of PD is approximately 60 years (Lees et al., 2009). However, a few patients are diagnosed before the age of 50 years, who are defined as those with early onset PD (EOPD), and those diagnosed with PD from 21 to 40 or 50 years of age are typically classified as young-onset PD (YOPD) (Wickremaratchi et al., 2009; Ferguson et al., 2016; Parkinson’s Disease and Movement Disorders Group of Neurology Branch of Chinese Medical Association and Parkinson’s Disease and Movement Disorders Group of Neurologist Branch of Chinese Physician Association, 2021).

The characteristics of patients with YOPD differ from those of older adults with PD (Wickremaratchi et al., 2009). Even with slow progression, patients with YOPD usually have a longer disease duration, and a more than five-fold higher mortality compared to a general population (Mehanna and Jankovic, 2019; Hustad et al., 2021). Due to the specific age of onset, patients with YOPD also tend to face a certain set of physical, economic, and psychological challenges and show poor social cognition (Calne et al., 2008; Wielinski et al., 2010; Mehanna and Jankovic, 2019; Seubert-Ravelo et al., 2021). In addition, genetic mutations are believed to contribute more toward the aetiology of YOPD than of late-onset PD (Rissling et al., 2009; Klepac et al., 2013).

Pharmacotherapy is one of the main treatment options for patients with YOPD (Klepac et al., 2013). The type of drug to be administered for patients with YOPD depends on multiple factors, and levodopa is commonly recognised as the most effective treatment (Klepac et al., 2013). Even so, there are no specific pharmacotherapy-related guidelines for patients with YOPD. In China, it is recommended that pharmacotherapy for patients with YOPD should be similar to that for patients with general PD [(Parkinson’s Disease and Movement Disorders Group of Neurology Branch of Chinese Medical Association and Parkinson’s Disease and Movement Disorders Group of Neurologist Branch of Chinese Physician Association, 2021). The special characteristics of patients with YOPD may result in different pharmacological patterns from those with PD, but it is unclear till now with few literature reports (Kasamo et al., 2019).

YOPD is a rare disease not only in China, but all over the world, with a prevalence of lower than 0.1‰ (Marras et al., 2018; GBD Neurology Collaborators, 2019; He et al., 2019; Park et al., 2019). A higher percentage of YOPD patients in Asians was previously reported compared to other ethnic groups, which may be accounted by the genetic susceptibility (Van Den Eeden et al., 2003; Post et al., 2020). It is easy to speculate that the prevalence of YOPD in China is high due to the similar ethnic traits, although there are no relevant reports. To date, a few studies have described the pharmacological treatment patterns for patients with YOPD in the real-world setting; however, those studies had a limited sample size (Kasamo et al., 2019; Camerucci et al., 2021). The foreseeable larger-sample of YOPD patients in China may provide key information about the prescription pattern in YOPD patients, which may help the administrators and clinicians to understand the situation, identify potential problems and promote medical optimization.

Therefore, in this study, we aimed to conduct a national large-scale study to describe the patterns of prescriptions for patients with YOPD in a real-world setting in China.

## Methods

### Data Source

This study was a subgroup analysis of ‘The program of multi-centre real-word comprehensive study of anti-PD drugs in China.’ All data were obtained from 30 hospitals in Anhui, Beijing, Guangdong, Henan, Jiangsu, Shandong, Shaanxi, Shanghai, Sichuan, Tianjin, and Zhejiang. Outpatient prescription data with information on the diagnosis, including that of PD, parkinsonism, and paralysis agitans, were extracted from the hospital information system using a random number table from 1 January 2014 to 31 December 2019.

The prescription data were included for analysis if the prescribed drugs included either L-DOPA combinations (L-DOPA/benserazide, L-DOPA/carbidopa), dopamine agonists (DAs, pramipexole, ropinirole, piribedil, bromocriptine), catechol-O-methyl transferase (COMT) inhibitors (entacapone), monoamine oxidase B (MAO-B) inhibitors (selegiline, rasagiline), anticholinergics (trihexyphenidyl), or amantadine, which are all available in China mainland. Other approved anti-PD drugs, including rotigotine, diphenhydramine and so on, were rarely used in China and not included in the analysis. Patient records were excluded if there were no prescription data available.

Information extracted from the medical records included 1) medical information: visiting hospital, patient ID, age, sex, visiting time, prescription code, diagnosis, comorbid diseases; and 2) drug information: drug code, commodity name, generic name, specification, route of administration, dosage, unit price, frequency of administration, single dosage, etc.

According to the definition of YOPD in China (Parkinson’s Disease and Movement Disorders Group of Neurology Branch of Chinese Medical Association and Parkinson’s Disease and Movement Disorders Group of Neurologist Branch of Chinese Physician Association, 2021), patients aged 21–50 years were defined as having YOPD and were included for further analyses in this study. The patients were identified by their unique ID, and the number of patients was counted based on the year with their unique ID. All extracted data were used to describe the prescription pattern of anti-PD drugs. Moreover, a subgroup analysis was performed based on sex, age and geographical area.

The levodopa equivalent daily dose (LED) was calculated for each anti-PD drug according to the conversion factors (Tomlinson et al., 2010). The conversion factor was 0.75 for L-DOPA/carbidopa, 1 for L-DOPA/benserazide, 100 for pramipexole, 20 for ropinirole, 1 for piribedil, 10 for bromocriptine, 1.33 for entacapone, 10 for selegiline, and 1 for amantadine. All prescription records over the 6 years were included in the calculation of LED.

### Ethics

The study protocol was approved by the Ethics Committee of Beijing Tiantan Hospital, Capital Medical University (KY 2020-077-01). The requirement for informed consent from the participants was waived because the data were analysed anonymously.

### Statistical Analysis

Statistical analysis was conducted using R software (version 4.1.1, R Foundation for Statistical Computing, Vienna, Austria). Continuous variables are shown as mean ± standard deviation. Categorical variables are presented as numbers and percentages. Demographic and prescription information was grouped into counts.

A scatterplot of LED (mg) *versus* the study duration and the 95% confidence interval was plotted and calculated using *the ggplot2* package (version 3.3.5, https://cran.r-project.org/web/packages/ggplot2/index.html) in R software. In addition, the median (Q1–Q3) values of LED in the year-interval were calculated separately.

## Results

### Demographic Characteristics of Patients

In ‘The program of multi-centre real-word comprehensive study of anti-PD drugs in China’, a total of 96,422 prescription records were extracted from 8,420 outpatients. Among them, 1,134 patients aged 21-50 years, accounting for 13.5% of the PD cohort. The demographic characteristics of patients with YOPD in the year-interval are shown in Table 1. Males accounted for 53.6% of all patients with YOPD. In each year, from 2014 to 2019, patients with YOPD aged 41–50 years accounted for the largest proportion (67–79%). Mental and behavioural disorders and nervous system diseases were the most common comorbidities, with depression/anxiety being the most frequent one.

**TABLE 1:** Demographic characteristics of patients with young-onset Parkinson’s disease from 2014 to 2019 based on each year.

Characteristics	Year					
	2014 (*n* = 197)	2015 (*n* = 214)	2016 (*n* = 225)	2017 (*n* = 249)	2018 (*n* = 296)	2019 (*n* = 299)
Gender						
Male	93	106	100	123	146	125
Female	104	108	125	126	150	172
Age group (years)						
21–30	20	20	23	24	40	26
31–40	22	46	43	45	59	55
41–50	155	148	159	180	197	216
Comorbid diseases						
Mental and behavioral disorders	12	15	16	14	23	21
Depression/anxiety	11	13	16	13	21	20
Psychiatric symptoms	1	2	0	1	2	1
Nervous system diseases	21	19	18	24	21	17
Dementia/cognitive disorder	0	0	1	2	1	2
Insomnia/sleep disorders	11	9	11	15	17	11
Psychoneurosis	8	6	5	4	2	2
Headache/neurodynia	2	4	0	1	1	2
Neuropathy^a^	0	0	1	2	0	0
Autonomic dysfunction	0	0	0	2	2	4
Constipation	0	0	0	1	2	4
Autonomic nervous disorders	0	0	0	1	0	0
Limb pain	1	1	1	0	4	1

a Notes: Classification of neuropathy could not be specifically determined according to the medical information.

### Prescription Pattern of Anti-PD Drugs

**FIGURE 1:**
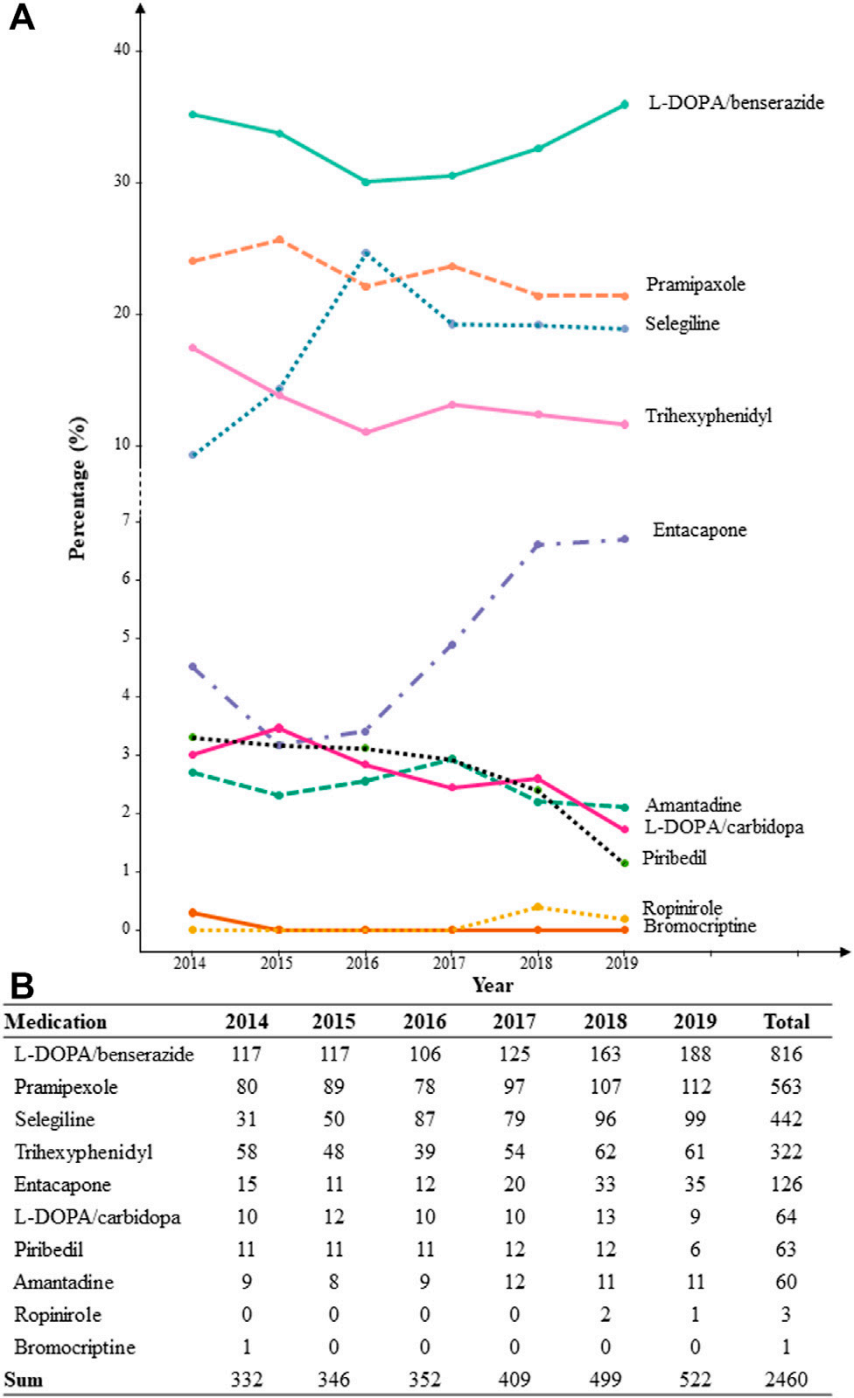
The percentage **(A)** and count **(B)** of patients with each prescribed anti-Parkinson’s disease drug from 2014 to 2019 based on each year.

Ten types of anti-PD drugs were identified as monotherapy or polytherapy in the population, namely, L-DOPA/benserazide, L-DOPA/carbidopa, pramipexole, ropinirole, piribedil, bromocriptine, entacapone, selegiline, trihexyphenidyl, and amantadine. In all prescriptions, about 86%–95% were prescribed by neurologists. The percentage of patients who were prescribed each type of anti-PD drug based on the year, from 2014 to 2019, is shown in Figure 1. L-DOPA/benserazide was the most commonly prescribed anti-PD drug in the past 6 years, with an average percentage of over 30% for all anti-PD drugs. Pramipexole, selegiline, and trihexyphenidyl, followed by L-DOPA/benserazide, accounted for over 10% of all anti-PD drugs. Other drugs were prescribed in < 10% of patients with YOPD each year. Similarly, L-DOPA/benserazide (36%), pramipexole (23%), selegiline (18%) and trihexyphenidyl (14%) were also the most commonly prescribed for these patients at their first medical visit.

**FIGURE 2:**
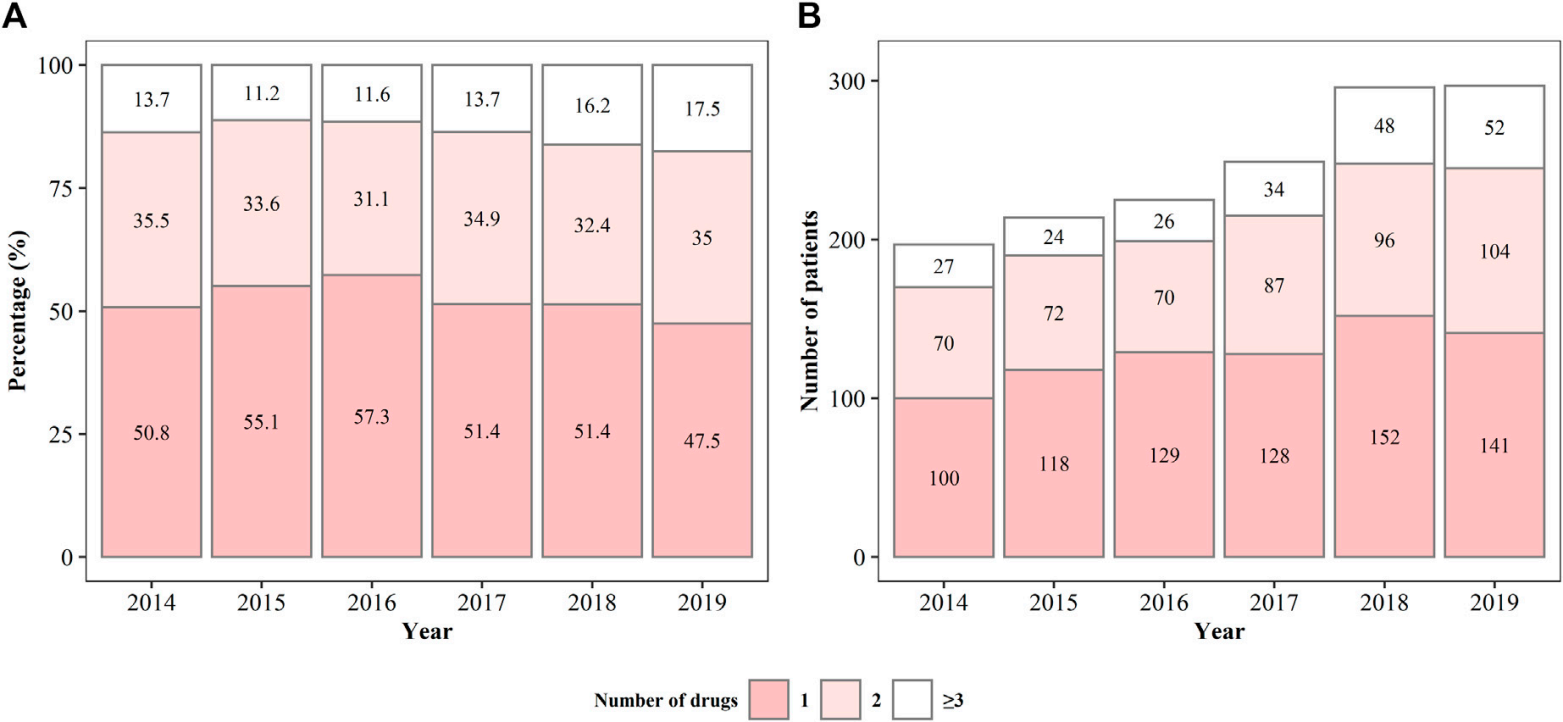
The percentage **(A)** and number **(B)** of patients being prescribed different anti-Parkinson’s disease drugs from 2014 to 2019.

The prescription patterns of anti-PD drugs are shown in Figure 2. From 2014 to 2019, approximately half of the patients with YOPD were on monotherapy; however, the percentage of patients on monotherapy showed a gradually decreasing trend from 2016 (57.3%) to 2019 (47.5%). Approximately one-third of the patients were treated with two anti-PD drugs, and the percentage remained almost unchanged over the 6 years. Correspondingly, the percentage of patients prescribed more than two anti-PD drugs gradually increased from 2015 (11.2%) to 2019 (17.5%). As the total number of patients with YOPD has increased over the past 6 years, the number of patients with YOPD receiving monotherapy or polytherapy has also increased. There were one or two patients prescribed with more than four drugs each year, and the maximum number of prescribed anti-PD drugs was seven (Supplementary Table S1).

**FIGURE 3:**
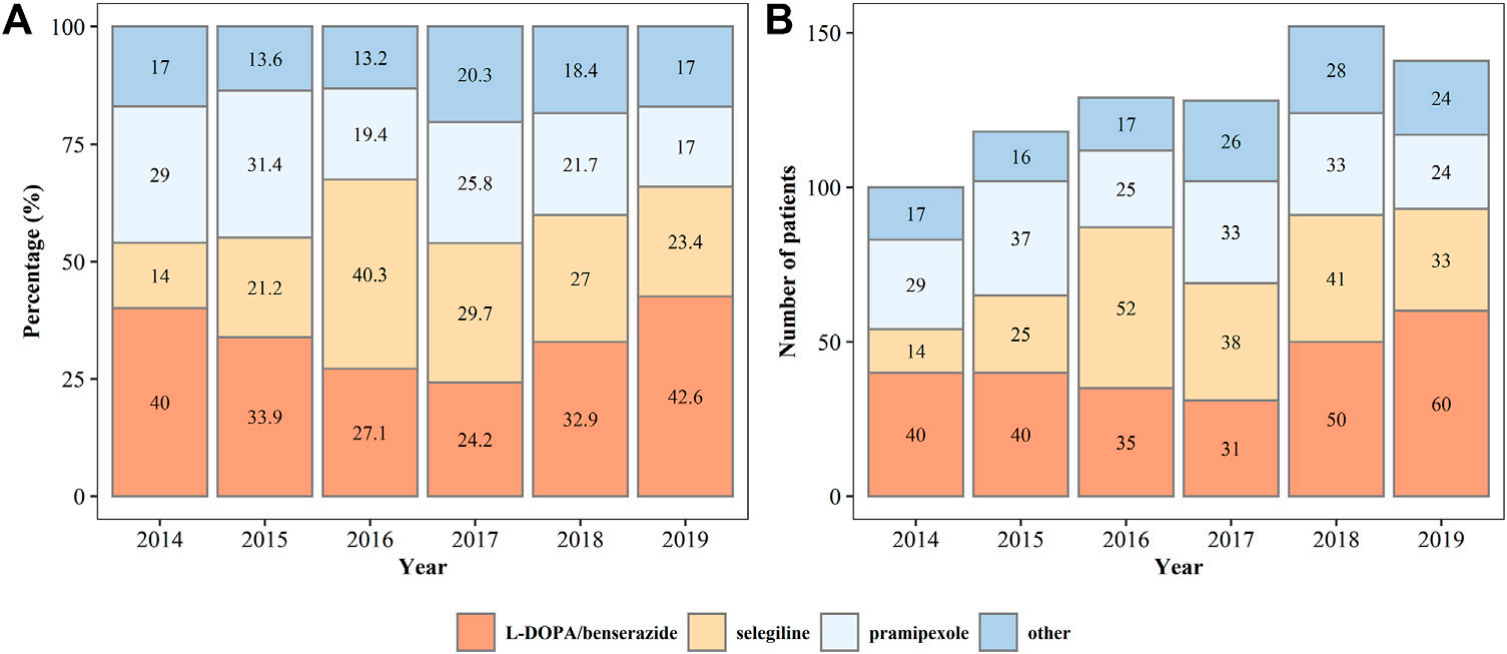
The percentage **(A)** and number **(B)** of patients on monotherapy of anti-Parkinson’s disease drugs from 2014 to 2019.

**FIGURE 4:**
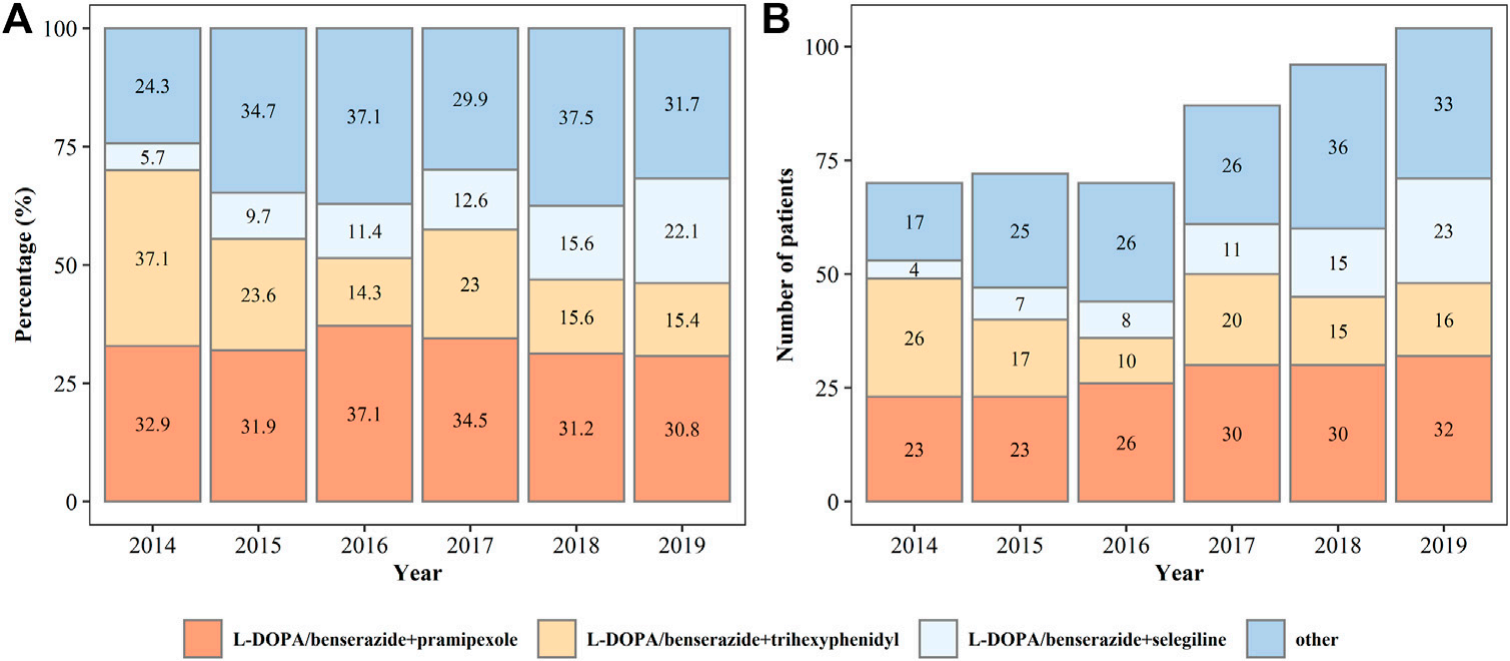
The percentage **(A)** and number **(B)** of patients being prescribed two types of anti-Parkinson’s disease drugs from 2014 to 2019.

In all patients on monotherapy, L-DOPA/benserazide, pramipexole, and selegiline were the most commonly prescribed drugs (Figure 3), accounting for more than 80% of the prescriptions. The percentage and number of patients on monotherapy with L-DOPA/benserazide or selegiline changed dynamically from 2014 to 2019. In patients prescribed two drugs, L-DOPA/benserazide combined with another anti-PD drug was the most common choice, especially with pramipexole (Figure 4). The combination of L-DOPA/benserazide and trihexyphenidyl was the second most commonly prescribed, but the percentage of patients on this type of polytherapy was decreasing.

In terms of sex and age, there was no difference in the prescribing patterns among different subgroups. Compared to that in the two cities with optimal medical level in China (Beijing and Shanghai), prescription patterns in other regions did not show difference, either.

### Relationship Between LED and Disease Duration

**FIGURE 5:**
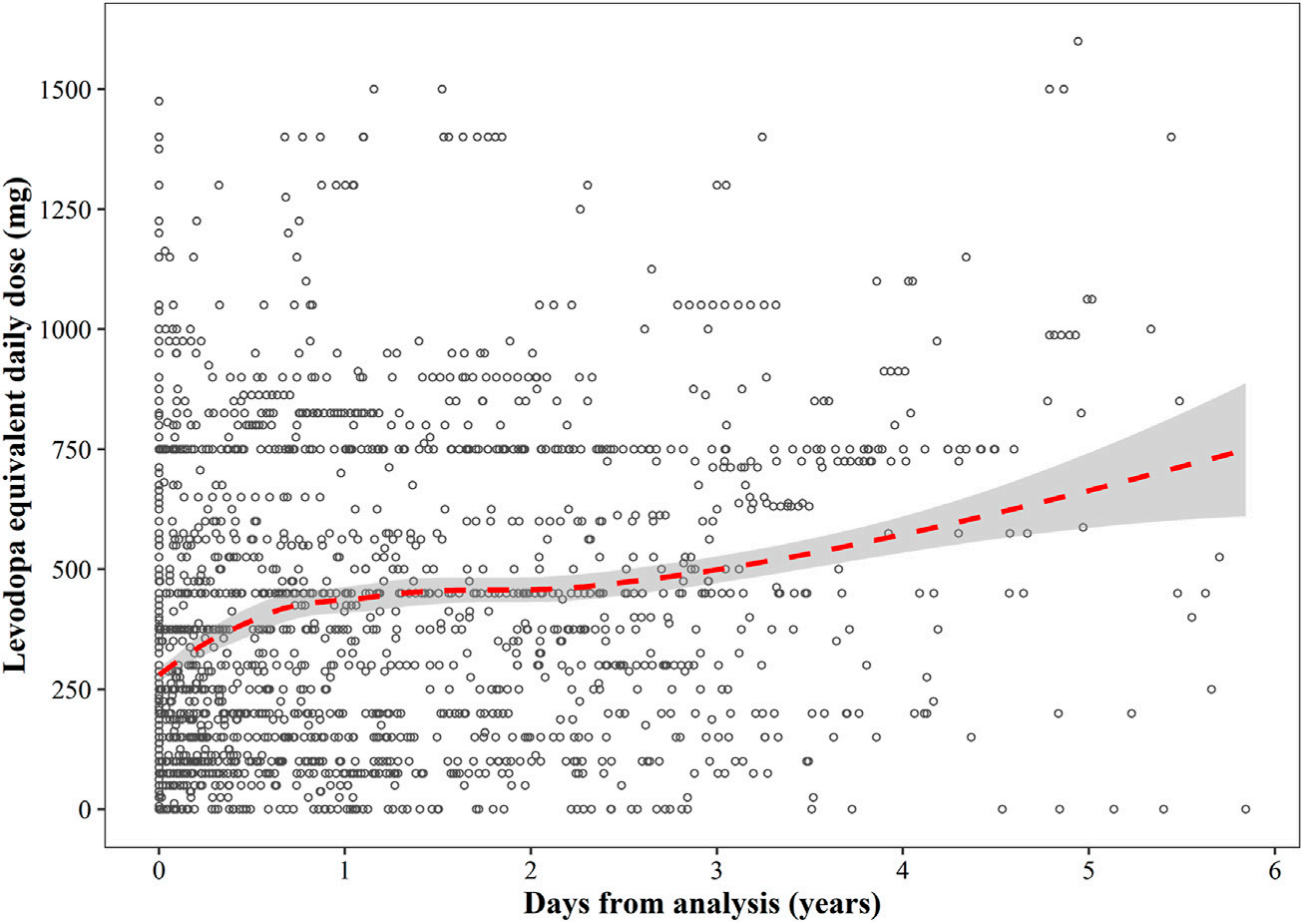
A scatterplot of the relationship between levodopa equivalent daily dose and disease duration. The red dotted line represents the regression curve, and the grey shaded area represents 95% confidence interval of the regression line.

The follow-up period ranged from 0 to 2,133 days from diagnosis, with a median disease duration of 2.7 years. The relationship between LED (mg/day) and disease duration is shown in Figure 5. LED increased along with the days from analysis as a near linear mode in the first year of analysis, followed by a relatively flat trend in the next 2 years. After 3 years from analysis, LED once again increased along with time.

A large variability in LED was shown at the beginning, ranging from 0 to 1,500 mg/day. The median (Q1–Q3) LED at 1-year intervals was 250 (150–750) mg/day at 0–1 year, 418.75 (150–750) mg/day at 1–2 years, 450 (250–612.5) mg/day at 2–3 years, 631.25 (300–750) mg/day at 3–4 years, and 750 (400–975) mg/day at > 4 years.

## Discussion

To the best of our knowledge, this is the first nationwide and large-scale investigation that aimed to describe the real-world patterns of prescription of patients with YOPD in China. L-DOPA combinations and DAs were the most commonly prescribed medications in this cohort of 1,134 patients with YOPD from different regions of China. Half of the patients with YOPD were on monotherapy; however, this percentage gradually declined. A gradually increasing trend was observed between LED and disease duration.

In our study, the proportion of male patients was higher than that of female patients (53.6% *versus* 46.4%), and those aged 40–50 years accounted for the majority of patients, which was consistent with the findings of previous studies (Mehanna et al., 2014; Kasamo et al., 2019; Camerucci et al., 2021). This could be explained by the gradual decline in neurological function over time and disease progression. In addition, partly because the diagnosis of YOPD is generally based on clinical symptoms, the overall accuracy of clinical diagnosis is lower in EOPD than in later stages of PD (Klepac et al., 2013). Compared to patients with late-onset PD, those with YOPD have fewer comorbidities (Klepac et al., 2013). The most common comorbidities in Chinese patients with YOPD are depression/anxiety and insomnia or sleep disorders, consistent with the findings of a previous report (Willis et al., 2013).

L-DOPA/benserazide was the most commonly prescribed anti-PD drug for patients with YOPD (55.2%), followed by pramipexole (38.1%) and selegiline (29.9%), in this study. Overall, the prescription pattern illustrated in the study was in accordance with the recommendations of the ‘Guidelines for treatment of Parkinson’s disease in China (2nd edition, 3rd edition)’ (Chen and Parkinson’s Disease and Movement Disorders Group of Neurology Branch of Chinese Medical Association, 2009; Parkinson’s Disease and Movement Disorders Group of Neurology Branch of Chinese Medical Association, 2014), which recommended L-DOPA combinations, COMT inhibitors, central DAs, anticholinergic drugs, MAO-B inhibitors, and amantadine for patients with PD, but without a specific recommendation for patients with YOPD. In 2019, ‘Chinese Expert Consensus on Diagnoses and Treatments of Early-onset Parkinson’s Disease’ was issued, and the principles of medication therapy mainly referred to the newly-issued ‘Guidelines for treatment of Parkinson’s disease in China (4th edition)’ published in 2020 (Parkinson’s Disease and Movement Disorders Group of Neurology Branch of Chinese Medical Association and Parkinson’s Disease and Movement Disorders Group of Neurologist Branch of Chinese Physician Association, 2020). The latter consensus declared that patients with YOPD show a good response to L-DOPA combinations, and L-DOPA combinations are also the priority drug for patients with YOPD having intellect impairment.

The prescription pattern of L-DOPA combinations in China was partly different from that in other countries. In China, the percentage of YOPD-prescribed L-DOPA/carbidopa was much lower than that of L-DOPA/benserazide (< 5% *versus* > 30% each year). A retrospective review in the United States reported that L-DOPA/carbidopa was the most frequent initial choice of medication for patients aged < 49 years (51.7%) (Mehanna et al., 2014). The proportion of Japanese patients with YOPD who were prescribed L-DOPA/carbidopa was also higher than those prescribed L-DOPA/benserazide (21.4% *versus* 9.2%) (Kasamo et al., 2019). In addition, a study in Taiwan district of China reported that less than 10% of patients with PD aged < 40 years were prescribed L-DOPA or DAs (Guo et al., 2014). A large variability in the choice of anti-PD drugs for young patients has been shown in different countries and regions.

The difference in prescription patterns of L-DOPA combinations could be explained by the fact that L-DOPA/carbidopa has been heavily out of stock in China in the past few years because of the shortage of drugs (American Society of Hospital Pharmacists, 2020). In addition, the Generic Drug Consistency Evaluation (GDCE), which aims to test the bioequivalence of domestic generic drugs compared with branded drugs, has been mandatory for drug manufacturers in China since 2015 (Wang et al., 2019). However, no generic drug of L-DOPA/carbidopa has passed the GDCE as of December 2021. A shortage of branded drugs and lack of generic drugs led to the transition from L-DOPA/carbidopa to L-DOPA/benserazide, or other DAs.

DAs are also commonly prescribed for patients with YOPD in China. Some evidence has shown that a lower risk of developing motor complications and dyskinesia, along with motor fluctuations in long-term treatment, was associated with the use of DAs rather than levodopa (Rascol et al., 2006; Stowe et al., 2010). Pramipexole was the most commonly prescribed DA, accounting for approximately 90% of all DAs. Non-ergot DAs have been suggested to effectively control movement disorder and are more appropriate for patients with YOPD in the initial period of disease (Fox et al., 2018; Parkinson’s Disease and Movement Disorders Group of Neurology Branch of Chinese Medical Association and Parkinson’s Disease and Movement Disorders Group of Neurologist Branch of Chinese Physician Association, 2021). Other non-ergot DAs, such as piribedil, ropinirole, and rotigotine, have been less frequently prescribed in China in our study.

The special prescription patterns for DAs may also be accounted for by the GDCE and the National Centralised Drug Procurement Policy in China. There were 10 generic drugs of pramipexole that passed the GDCE and were approved in China until November 2021, which ranked first among all anti-PD drugs. In addition, the National Centralised Drug Procurement Policy, which aims to reduce drug costs, has been implemented since January 2019 (Chen et al., 2020; Wang N et al., 2021). Therefore, multiple selectivity of generic drugs of pramipexole and lower drug cost induced by the National Centralised Drug Procurement Policy may together account for the higher proportion of patients with YOPD who are prescribed pramipexole compared to other DAs.

In our study, approximately half of the patients with YOPD were on monotherapy and approximately 30% of patients were on dual drug combination therapy. This may indicate a good response to medication in most patients with YOPD (Klepac et al., 2013). However, it was also observed that the proportion of patients receiving monotherapy decreased from 2016 to 2019, while the proportion of patients receiving polytherapy increased. Recent studies have demonstrated that the combination of levodopa and another anti-PD drugs was superior to levodopa monotherapy for the improvement of clinical symptoms and safety (Jiang et al., 2020; Jiang et al., 2021; Wang Y et al., 2021). The transition from monotherapy to polytherapy demonstrated in our study also indicates alterations in therapeutic strategies.

In this study, a gradually increasing trend was observed in the relationship between LED and disease duration. It is well known that the response to levodopa is good in patients with YOPD in the initial period of pharmacotherapy but gradually becomes resistant as the treatment progresses (Quinn et al., 1987; Klepac et al., 2013). The trend of LED in our study was similar to that reported in newly diagnosed Japanese patients with YOPD, but an overall higher median LED was observed in our study, as compared to that in Japanese individuals (Kasamo et al., 2019). This was because our study included not only newly diagnosed patients with YOPD but also some patients who were administered anti-PD pharmacotherapy when they were included in the analysis. This indicated that the increasing trend in LED over time was common for both newly diagnosed and already diagnosed patients with YOPD.

Our study has some limitations. First, we could not exclude patients who were diagnosed with drug-induced PD from the total population. A better drug response may be achieved in these patients once the causes of the disease have been excluded. Second, the majority of the study population was from economically developed regions in China. The prescription patterns in some underdeveloped areas may be different due to discrepancies in access to medical and economic factors. Third, we did not collect data regarding the proportion of patients with motor fluctuations, and medication strategies for the management of motor fluctuations, which limited further analysis about the diverse selection of medication along with disease duration. Forth, due to lack of information about disease severity, our data did not support a subgroup analysis to explore prescription difference among patients with varying degrees of disease severity.

## Conclusion

In conclusion, using a national multi-centre data source, we comprehensively described the prescription patterns of anti-PD drugs for patients with YOPD in China over a 6-years period. L-DOPA combinations and non-ergot DAs were the most frequently prescribed anti-PD drugs. Approximately half of the patients were on monotherapy, but the proportion of patients receiving polytherapy was increasing. In addition, LED and disease duration showed a gradually increasing non-linear trend. Overall, our study provides a general overview of the pharmacological treatment pattern of anti-PD drugs in patients with YOPD and may be beneficial for the clinical management of YOPD.

## Data Availability

The original contributions presented in the study are included in the article/Supplementary Material, further inquiries can be directed to the corresponding author.

## References

[B1] American Society of Hospital Pharmacists (2020). E. coli. Available at: https://www.ashp.org/drug- shortages/current-shortages/drug-shortage-detail.aspx?id=349 (Accessed Dec 31, 2021).

[B2] Armstrong M. J. Okun M. S. (2020). Diagnosis and Treatment of Parkinson Disease: A Review. JAMA 323, 548–560. 10.1001/jama.2019.22360 32044947

[B3] Calabrese V. P. (2007). Projected Number of People with Parkinson Disease in the Most Populous Nations, 2005 through 2030. Neurology 69, 223–224. author reply 224. 10.1212/01.wnl.0000271777.50910.73 17620562

[B4] Calne S. M. Lidstone S. C. Kumar A. (2008). Psychosocial Issues in Young-Onset Parkinson's Disease: Current Research and Challenges. Parkinsonism Relat. Disord. 14, 143–150. 10.1016/j.parkreldis.2007.07.012 17889588

[B5] Camerucci E. Stang C. D. Hajeb M. Turcano P. Mullan A. F. Martin P. (2021). Early-Onset Parkinsonism and Early-Onset Parkinson's Disease: A Population-Based Study (2010-2015). J. Parkinsons Dis. 11, 1197–1207. 10.3233/JPD-202464 33720851 PMC8355040

[B6] Chen L. Yang Y. Luo M. Hu B. Yin S. Mao Z. (2020). The Impacts of National Centralized Drug Procurement Policy on Drug Utilization and Drug Expenditures: The Case of Shenzhen, China. Ijerph 17, 9415. 10.3390/ijerph17249415 PMC7765443 33334027

[B7] Chen S. D. Parkinson's Disease and Movement Disorders Group of Neurology Branch of Chinese Medical Association (2009). Chinese Guidelines for the Treatment of Parkinson′s Disease (Second Edition). Chin. J. Neurol. 42, 352–355. 10.3760/cma.j

[B8] Ferguson L. W. Rajput A. H. Rajput A. (2016). Early-onset vs. Late-Onset Parkinson's Disease: A Clinical-Pathological Study. Can. J. Neurol. Sci. 43, 113–119. 10.1017/cjn.2015.244 26189779

[B9] Fox S. H. Katzenschlager R. Lim S. Y. Barton B. De Bie R. M. A. Seppi K. (2018). International Parkinson and Movement Disorder Society Evidence-Based Medicine Review: Update on Treatments for the Motor Symptoms of Parkinson's Disease. Mov Disord. 33, 1248–1266. 10.1002/mds.27372 29570866

[B10] GBD Neurology Collaborators (2019). Global, Regional, and National burden of Neurological Disorders, 1990-2016: a Systematic Analysis for the Global Burden of Disease Study 2016. Lancet Neurol. 18, 459–480. 10.1016/S1474-4422(18)30499-X 30879893 PMC6459001

[B11] Guo Y. J. Liao Y. C. Lin C. H. Chang M. H. (2014). Initial Medication in Patients of Newly Diagnosed Parkinson's Disease in Taiwan. PLoS One 9, e107465. 10.1371/journal.pone.0107465 25222829 PMC4164642

[B12] He J. Tang M. Zhang X. Chen D. Kang Q. Yang Y. (2019). Incidence and Prevalence of 121 Rare Diseases in China: Current Status and Challenges. Intractable Rare Dis. Res. 8, 89–97. 10.5582/irdr.2019.01066 31218158 PMC6557238

[B13] Hustad E. Myklebust T. Å. Gulati S. Aasly J. O. (2021). Increased Mortality in Young-Onset Parkinson's Disease. J. Mov Disord. 14, 214–220. 10.14802/jmd.21029 34315208 PMC8490197

[B14] Jiang D. Q. Li M. X. Jiang L. L. Chen X. B. Zhou X. W. (2020). Comparison of Selegiline and Levodopa Combination Therapy versus Levodopa Monotherapy in the Treatment of Parkinson's Disease: a Meta-Analysis. Aging Clin. Exp. Res. 32, 769–779. 10.1007/s40520-019-01232-4 31175606

[B15] Jiang D. Q. Zang Q. M. Jiang L. L. Wang Y. Li M. X. Qiao J. Y. (2021). Comparison of Pramipexole and Levodopa/benserazide Combination Therapy versus Levodopa/benserazide Monotherapy in the Treatment of Parkinson's Disease: a Systematic Review and Meta-Analysis. Naunyn Schmiedebergs Arch. Pharmacol. 394, 1893–1905. 10.1007/s00210-021-02089-z 33959780

[B16] Kasamo S. Takeuchi M. Ikuno M. Kawasaki Y. Tanaka S. Takahashi R. (2019). Real-world Pharmacological Treatment Patterns of Patients with Young-Onset Parkinson's Disease in Japan: a Medical Claims Database Analysis. J. Neurol. 266, 1944–1952. 10.1007/s00415-019-09360-7 31076875

[B17] Klepac N. Habek M. Adamec I. Barušić A. K. Bach I. Margetić E. (2013). An Update on the Management of Young-Onset Parkinson's Disease. Degener Neurol. Neuromuscul. Dis. 2, 53–62. 10.2147/dnnd.S34251 30890879 PMC6065598

[B18] Lees A. J. Hardy J. Revesz T. (2009). Parkinson's Disease. Lancet 373, 2055–2066. 10.1016/S0140-6736(09)60492-X 19524782

[B19] Li G. Ma J. Cui S. He Y. Xiao Q. Liu J. (2019). Parkinson's Disease in China: a Forty-Year Growing Track of Bedside Work. Transl Neurodegener 8, 22. 10.1186/s40035-019-0162-z 31384434 PMC6668186

[B20] Marras C. Beck J. C. Bower J. H. Roberts E. Ritz B. Ross G. W. (2018). Prevalence of Parkinson's Disease across North America. NPJ Parkinsons Dis. 4, 21. 10.1038/s41531-018-0058-0 30003140 PMC6039505

[B21] Mehanna R. Jankovic J. (2019). Young-onset Parkinson's Disease: Its Unique Features and Their Impact on Quality of Life. Parkinsonism Relat. Disord. 65, 39–48. 10.1016/j.parkreldis.2019.06.001 31176633

[B22] Mehanna R. Moore S. Hou J. G. Sarwar A. I. Lai E. C. (2014). Comparing Clinical Features of Young Onset, Middle Onset and Late Onset Parkinson's Disease. Parkinsonism Relat. Disord. 20, 530–534. 10.1016/j.parkreldis.2014.02.013 24631501

[B23] Park J. H. Kim D. H. Kwon D. Y. Choi M. Kim S. Jung J. H. (2019). Trends in the Incidence and Prevalence of Parkinson's Disease in Korea: a Nationwide, Population-Based Study. BMC Geriatr. 19, 320. 10.1186/s12877-019-1332-7 31752705 PMC6868716

[B24] Parkinson's Disease and Movement Disorders Group of Neurology Branch of Chinese Medical Association (2014). Chinese Guidelines for the Treatment of Parkinson′s Disease (Third Edition). Chin. J. Neurol. 47, 428–433. 10.3760/cma.j.issn.1006-7876.2014.06.017

[B25] Parkinson's Disease and Movement Disorders Group of Neurology Branch of Chinese Medical Association, and Parkinson's Disease and Movement Disorders Group of Neurologist Branch of Chinese Physician Association (2021). Chinese Expert Consensus on Diagnoses and Treatments of Early-Onset Parkinson's Disease. Chin. J. Neuromed 20, 109–116. (in Chinese). 10.3760/cma.j.cn115354-20201119-00903

[B26] Parkinson's Disease and Movement Disorders Group of Neurology Branch of Chinese Medical Association, and Parkinson's Disease and Movement Disorders Group of Neurologist Branch of Chinese Physician Association (2020). Chinese Guidelines for the Treatment of Parkinson′s Disease (Fourth Edition). Chin. J. Neuromed 53, 973–986. 10.3760/cma.j.cn113694-20200331-00233

[B27] Post B. Van Den Heuvel L. Van Prooije T. Van Ruissen X. Van De Warrenburg B. Nonnekes J. (2020). Young Onset Parkinson's Disease: A Modern and Tailored Approach. J. Parkinsons Dis. 10, S29–s36. 10.3233/jpd-202135 32651336 PMC7592661

[B28] Quinn N. Critchley P. Marsden C. D. (1987). Young Onset Parkinson's Disease. Mov Disord. 2, 73–91. 10.1002/mds.870020201 3504266

[B29] Rascol O. Brooks D. J. Korczyn A. D. De Deyn P. P. Clarke C. E. Lang A. E. (2006). Development of Dyskinesias in a 5-year Trial of Ropinirole and L-Dopa. Mov Disord. 21, 1844–1850. 10.1002/mds.20988 16958094

[B30] Rissling I. Strauch K. Höft C. Oertel W. H. Möller J. C. (2009). Haplotype Analysis of the Engrailed-2 Gene in Young-Onset Parkinson's Disease. Neurodegener Dis. 6, 102–105. 10.1159/000207796 19270442

[B31] Seubert-Ravelo A. N. Yáñez-Téllez M. G. Lazo-Barriga M. L. Calderón Vallejo A. Martínez-Cortés C. E. Hernández-Galván A. (2021). Social Cognition in Patients with Early-Onset Parkinson's Disease. Parkinsons Dis. 2021, 8852087. 10.1155/2021/8852087 33505651 PMC7810525

[B32] Stowe R. Ives N. Clarke C. E. Deane K. Van H. Wheatley K. (2010). Evaluation of the Efficacy and Safety of Adjuvant Treatment to Levodopa Therapy in Parkinson S Disease Patients with Motor Complications. Cochrane Database Syst. Rev., CD007166. 10.1002/14651858.CD007166.pub2 20614454 PMC12832168

[B33] Tomlinson C. L. Stowe R. Patel S. Rick C. Gray R. Clarke C. E. (2010). Systematic Review of Levodopa Dose Equivalency Reporting in Parkinson's Disease. Mov Disord. 25, 2649–2653. 10.1002/mds.23429 21069833

[B34] Van Den Eeden S. K. Tanner C. M. Bernstein A. L. Fross R. D. Leimpeter A. Bloch D. A. (2003). Incidence of Parkinson's Disease: Variation by Age, Gender, and Race/ethnicity. Am. J. Epidemiol. 157, 1015–1022. 10.1093/aje/kwg068 12777365

[B35] Wang M. Ji Y. C. Liu J. X. Li K. Xie Z. W. Guo M. Y. (2019). Research on Progress of Generic Drug Consistency Evaluation in China. Chin. J. Drug Eval. 36, 460–463. (in Chinese).

[B36] Wang N. Yang Y. Xu L. Mao Z. Cui D. (2021). Influence of Chinese National Centralized Drug Procurement on the price of Policy-Related Drugs: an Interrupted Time Series Analysis. BMC Public Health 21, 1883. 10.1186/s12889-021-11882-7 34663282 PMC8524972

[B37] Wang Y. Jiang D.-Q. Lu C.-S. Li M.-X. Jiang L.-L. (2021). Efficacy and Safety of Combination Therapy with Pramipexole and Levodopa vs Levodopa Monotherapy in Patients with Parkinson Disease: A Systematic Review and Meta-Analysis. Medicine (Baltimore) 100, e27511. 10.1097/MD.0000000000027511 34871213 PMC8568447

[B38] Wickremaratchi M. M. Ben-Shlomo Y. Morris H. R. (2009). The Effect of Onset Age on the Clinical Features of Parkinson's Disease. Eur. J. Neurol. 16, 450–456. 10.1111/j.1468-1331.2008.02514.x 19187262

[B39] Wielinski C. L. Varpness S. C. Erickson-Davis C. Paraschos A. J. Parashos S. A. (2010). Sexual and Relationship Satisfaction Among Persons with Young-Onset Parkinson's Disease. J. Sex. Med. 7, 1438–1444. 10.1111/j.1743-6109.2009.01408.x 19656271

[B40] Willis A. W. Schootman M. Kung N. Racette B. A. (2013). Epidemiology and Neuropsychiatric Manifestations of Young Onset Parkinson's Disease in the United States. Parkinsonism Relat. Disord. 19, 202–206. 10.1016/j.parkreldis.2012.09.014 23083512 PMC3562561

[B41] Zou Y. M. Liu J. Tian Z. Y. Lu D. Zhou Y. Y. (2015). Systematic Review of the Prevalence and Incidence of Parkinson's Disease in the People's Republic of China. Neuropsychiatr. Dis. Treat. 11, 1467–1472. 10.2147/NDT.S85380 26109861 PMC4474453

