# Erratum: Real-World Prescription Patterns For Patients With Young-Onset Parkinson’s Disease in China: A Trend Analysis From 2014 to 2019

**DOI:** 10.3389/fphar.2022.972053

**Published:** 2022-07-21

**Authors:** Frontiers Production Office

**Affiliations:** Frontiers Media SA, Lausanne, Switzerland

**Keywords:** prescribing pattern, young-onset Parkinson’s disease, levodopa, dopamine agonist, levodopa equivalence daily dose

Due to a production error, Xiao-yu Wang was listed as the second author, instead of the co-first author.

The publisher apologizes for this mistake. The original version of this article has been updated.

