# Supplementary material for: Real-World Prescription Patterns For Patients With Young-Onset Parkinson’s Disease in China: A Trend Analysis From 2014 to 2019
Source: Front Pharmacol. 2022 May 12;13:858139. doi: 10.3389/fphar.2022.858139 (PMC9133339; doi:10.3389/fphar.2022.858139)
Supplement: Supplementary file 1 [file DataSheet1.DOCX]

# Supplementary Table 1. Distribution of all anti-Parkinson’s disease drugs prescribed from 2014 to 2019.

| **Number**  **of drugs** | **Drug name** | **Year** | | | | | | **Total** |
| --- | --- | --- | --- | --- | --- | --- | --- | --- |
|  |  | **2014** | **2015** | **2016** | **2017** | **2018** | **2019** |  |
| 1 |  | 100 | 118 | 129 | 128 | 152 | 141 | 768 |
|  | TRI | 11 | 9 | 9 | 5 | 8 | 5 | 47 |
|  | PIR | 1 | 1 | 0 | 4 | 1 | 0 | 7 |
|  | L-DOPA/BEN | 40 | 40 | 35 | 31 | 50 | 60 | 256 |
|  | ENT | 3 | 3 | 3 | 12 | 12 | 15 | 48 |
|  | AMA | 1 | 2 | 2 | 3 | 2 | 2 | 12 |
|  | L-DOPA/CAR | 0 | 1 | 3 | 2 | 4 | 2 | 12 |
|  | ROP | 0 | 0 | 0 | 0 | 1 | 0 | 1 |
|  | PRA | 29 | 37 | 25 | 33 | 33 | 24 | 181 |
|  | SEL | 14 | 25 | 52 | 38 | 41 | 33 | 203 |
|  | BRO | 1 | 0 | 0 | 0 | 0 | 0 | 1 |
| 2 |  | 70 | 72 | 70 | 87 | 96 | 104 | 499 |
|  | TRI+PIR | 0 | 0 | 0 | 1 | 0 | 0 | 1 |
|  | TRI+L-DOPA/BEN | 26 | 17 | 10 | 20 | 15 | 16 | 104 |
|  | TRI+AMA | 1 | 0 | 0 | 2 | 0 | 0 | 3 |
|  | TRI+PRA | 1 | 3 | 3 | 5 | 5 | 10 | 27 |
|  | TRI+SEL | 1 | 4 | 6 | 4 | 8 | 0 | 23 |
|  | PIR+L-DOPA/BEN | 2 | 5 | 4 | 2 | 4 | 2 | 19 |
|  | PIR+ENT | 0 | 0 | 2 | 0 | 1 | 1 | 4 |
|  | PIR+AMA | 0 | 0 | 1 | 0 | 0 | 1 | 2 |
|  | PIR+L-DOPA/CAR | 0 | 0 | 0 | 0 | 2 | 0 | 2 |
|  | PIR+PRA | 1 | 0 | 0 | 0 | 0 | 0 | 1 |
|  | L-DOPA/BEN+ENT | 1 | 1 | 0 | 1 | 3 | 2 | 8 |
|  | L-DOPA/BEN+AMA | 1 | 2 | 1 | 2 | 2 | 1 | 9 |
|  | L-DOPA/BEN+L-DOPA/CAR | 1 | 1 | 0 | 0 | 0 | 2 | 4 |
|  | L-DOPA/BEN+ROP | 0 | 0 | 0 | 0 | 0 | 1 | 1 |
|  | L-DOPA/BEN+PRA | 23 | 23 | 26 | 30 | 30 | 32 | 164 |
|  | L-DOPA/BEN+SEL | 4 | 7 | 8 | 11 | 15 | 23 | 68 |
|  | ENT+PRA | 1 | 3 | 2 | 0 | 4 | 3 | 13 |
|  | ENT+SEL | 2 | 0 | 1 | 1 | 1 | 1 | 6 |
|  | AMA+PRA | 0 | 0 | 0 | 1 | 0 | 0 | 1 |
|  | L-DOPA/CAR+PRA | 3 | 4 | 3 | 2 | 3 | 0 | 15 |
|  | PRA+SEL | 2 | 2 | 3 | 5 | 3 | 9 | 24 |
| 3 |  | 19 | 16 | 21 | 29 | 38 | 38 | 161 |
|  | TRI+PIR+L-DOPA/BEN | 0 | 0 | 0 | 0 | 0 | 1 | 1 |
|  | TRI+L-DOPA/BEN+ENT | 0 | 0 | 0 | 0 | 1 | 4 | 5 |
|  | TRI+L-DOPA/BEN+AMA | 1 | 0 | 0 | 1 | 0 | 0 | 2 |
|  | TRI+L-DOPA/BEN+PRA | 7 | 6 | 0 | 4 | 8 | 6 | 31 |
|  | TRI+L-DOPA/BEN+SEL | 2 | 3 | 6 | 7 | 11 | 10 | 39 |
|  | TRI+ENT+L-DOPA/CAR | 1 | 1 | 0 | 0 | 0 | 0 | 2 |
|  | TRI+ENT+PRA | 1 | 0 | 0 | 0 | 0 | 0 | 1 |
|  | TRI+ENT+SEL | 0 | 0 | 0 | 1 | 0 | 0 | 1 |

**(continues)**

**Supplementary Table 1 continued**

| **Number**  **of drugs** | **Drug name** | **Year** | | | | | | **Total** |
| --- | --- | --- | --- | --- | --- | --- | --- | --- |
|  |  | **2014** | **2015** | **2016** | **2017** | **2018** | **2019** |  |
| 3 | TRI+PRA+SEL | 1 | 0 | 1 | 2 | 2 | 2 | 8 |
|  | PIR+L-DOPA/BEN+ENT | 0 | 0 | 0 | 1 | 1 | 0 | 2 |
|  | PIR+L-DOPA/BEN+L-DOPA/CAR | 0 | 0 | 0 | 1 | 0 | 0 | 1 |
|  | PIR+L-DOPA/BEN+PRA | 0 | 0 | 1 | 1 | 0 | 0 | 2 |
|  | PIR+ENT+PRA | 1 | 0 | 0 | 0 | 0 | 0 | 1 |
|  | PIR+AMA+L-DOPA/CAR | 1 | 1 | 1 | 0 | 0 | 0 | 3 |
|  | L-DOPA/BEN+ENT+PRA | 1 | 0 | 1 | 0 | 4 | 2 | 8 |
|  | L-DOPA/BEN+ENT+SEL | 0 | 0 | 0 | 0 | 2 | 1 | 3 |
|  | L-DOPA/BEN+AMA+PRA | 0 | 0 | 1 | 0 | 1 | 2 | 4 |
|  | L-DOPA/BEN+L-DOPA/CAR+PRA | 0 | 0 | 1 | 2 | 0 | 1 | 4 |
|  | L-DOPA/BEN+PRA+SEL | 2 | 5 | 7 | 7 | 7 | 9 | 37 |
|  | ENT+AMA+L-DOPA/CAR | 0 | 0 | 1 | 1 | 0 | 0 | 2 |
|  | ENT+PRA+SEL | 0 | 0 | 1 | 0 | 0 | 0 | 1 |
|  | AMA+ROP+PRA | 0 | 0 | 0 | 0 | 1 | 0 | 1 |
|  | AMA+PRA+SEL | 0 | 0 | 0 | 1 | 0 | 0 | 1 |
|  | L-DOPA/CAR+PRA+SEL | 1 | 0 | 0 | 0 | 0 | 0 | 1 |
| 4 |  | 5 | 6 | 5 | 5 | 9 | 12 | 42 |
|  | TRI+PIR+L-DOPA/BEN+AMA | 0 | 0 | 1 | 0 | 0 | 0 | 1 |
|  | TRI+PIR+L-DOPA/BEN+PRA | 1 | 0 | 0 | 0 | 0 | 0 | 1 |
|  | TRI+L-DOPA/BEN+AMA+L-DOPA/CAR | 0 | 0 | 1 | 0 | 0 | 0 | 1 |
|  | TRI+L-DOPA/BEN+AMA+SEL | 0 | 0 | 0 | 0 | 0 | 1 | 1 |
|  | TRI+L-DOPA/BEN+PRA+SEL | 1 | 2 | 2 | 1 | 3 | 4 | 13 |
|  | TRI+ENT+AMA+L-DOPA/CAR | 0 | 1 | 0 | 0 | 0 | 0 | 1 |
|  | TRI+ENT+PRA+SEL | 0 | 0 | 0 | 1 | 0 | 0 | 1 |
|  | PIR+L-DOPA/BEN+ENT+PRA | 1 | 0 | 1 | 1 | 0 | 0 | 3 |
|  | PIR+L-DOPA/BEN+AMA+L-DOPA/CAR | 0 | 1 | 0 | 1 | 1 | 0 | 3 |
|  | PIR+L-DOPA/BEN+AMA+SEL | 0 | 0 | 0 | 0 | 2 | 0 | 2 |
|  | PIR+L-DOPA/BEN+L-DOPA/CAR+PRA | 0 | 1 | 0 | 0 | 0 | 0 | 1 |
|  | L-DOPA/BEN+ENT+AMA+L-DOPA/CAR | 0 | 0 | 0 | 0 | 1 | 0 | 1 |
|  | L-DOPA/BEN+ENT+L-DOPA/CAR+PRA | 0 | 0 | 0 | 1 | 1 | 1 | 3 |
|  | L-DOPA/BEN+ENT+PRA+SEL | 1 | 1 | 0 | 0 | 1 | 4 | 7 |
|  | L-DOPA/BEN+AMA+L-DOPA/CAR+PRA | 1 | 0 | 0 | 0 | 0 | 0 | 1 |
|  | L-DOPA/BEN+AMA+PRA+SEL | 0 | 0 | 0 | 0 | 0 | 1 | 1 |
|  | AMA+L-DOPA/CAR+PRA+SEL | 0 | 0 | 0 | 0 | 0 | 1 | 1 |

**(continues)**

**Supplementary Table 1 continued**

| **Number**  **of drugs** | **Drug name** | **Year** | | | | | | **Total** |
| --- | --- | --- | --- | --- | --- | --- | --- | --- |
|  |  | **2014** | **2015** | **2016** | **2017** | **2018** | **2019** |  |
| 5 |  | 3 | 1 | 0 | 0 | 1 | 1 | 6 |
|  | TRI+PIR+L-DOPA/BEN+ENT+PRA | 0 | 1 | 0 | 0 | 0 | 0 | 1 |
|  | TRI+PIR+L-DOPA/BEN+AMA+L-DOPA/CAR | 0 | 0 | 0 | 0 | 0 | 1 | 1 |
|  | TRI+PIR+L-DOPA/BEN+AMA+PRA | 1 | 0 | 0 | 0 | 0 | 0 | 1 |
|  | TRI+PIR+ENT+AMA+L-DOPA/CAR | 2 | 0 | 0 | 0 | 0 | 0 | 2 |
|  | TRI+ENT+AMA+L-DOPA/CAR+PRA | 0 | 0 | 0 | 0 | 1 | 0 | 1 |
| 6 |  | 0 | 0 | 0 | 0 | 0 | 1 | 1 |
|  | TRI+L-DOPA/BEN+ENT+AMA+L-DOPA/CAR+PRA | 0 | 0 | 0 | 0 | 0 | 1 | 1 |
| 7 |  | 0 | 1 | 0 | 0 | 0 | 0 | 1 |
|  | TRI+PIR+L-DOPA/BEN+AMA+L-DOPA/CAR+PRA+SEL | 0 | 1 | 0 | 0 | 0 | 0 | 1 |

**Abbreviation:** AMA, amantadine; BEN, benserazide; BRO, bromocriptine; CAR, carbidopa; ENT, entacapone; PIR, piribedil; PRA, pramipexole; ROP, ropinirole; SEL, selegiline; TRI, trihexyphenidyl.
